# Pragmatic randomized trial assessing the impact of digital health technology on quality of life in patients with heart failure: Design, rationale and implementation

**DOI:** 10.1002/clc.23848

**Published:** 2022-07-12

**Authors:** Angela M. Victoria‐Castro, Melissa Martin, Yu Yamamoto, Tariq Ahmad, Tanima Arora, Frida Calderon, Nihar Desai, Brett Gerber, Kyoung A. Lee, Daniel Jacoby, Hannah Melchinger, Andrew Nguyen, Melissa Shaw, Michael Simonov, Alyssa Williams, Jason Weinstein, Francis P. Wilson

**Affiliations:** ^1^ Clinical and Translational Research Accelerator (CTRA), Department of Medicine Yale University School of Medicine New Haven Connecticut USA; ^2^ Department of Medicine, Section of Cardiology Yale University School of Medicine New Haven Connecticut USA; ^3^ Department of Medicine, Section of Rheumatology, Allergy, and Immunology Yale University School of Medicine New Haven Connecticut USA; ^4^ Department of Medicine, Section of Nephrology Yale University School of Medicine New Haven Connecticut USA

**Keywords:** digital health technology, heart failure

## Abstract

**Background:**

Self‐care and patient engagement are important elements of heart failure (HF) care, endorsed in the guidelines. Digital health tools may improve quality of life (QOL) in HF patients by promoting care, knowledge, and engagement. This manuscript describes the rationale and challenges of the design and implementation of a pragmatic randomized controlled trial to evaluate the efficacy of three digital health technologies in improving QOL for patients with HF.

**Hypothesis:**

We hypothesize that digital health interventions will improve QOL of HF patients through the early detection of warning signs of disease exacerbation, the opportunity of self‐tracking symptoms, and the education provided, which enhances patient empowerment.

**Methods:**

Using a fully electronic enrollment and consent platform, the trial will randomize 200 patients across HF clinics in the Yale New Haven Health system to receive either usual care or one of three digital technologies designed to promote self‐management and provide critical data to clinicians. The primary outcome is the change in QOL as assessed by the Kansas City Cardiomyopathy Questionnaire at 3 months.

**Results:**

First enrollment occurred in September 2021. Recruitment was anticipated to last 6–8 months and participants were followed for 6 months after randomization. Our recruitment efforts have highlighted the large digital divide in our population of interest.

**Conclusion:**

Assessing clinical outcomes, patient usability, and ease of clinical integration of digital technologies will be beneficial in determining the feasibility of the integration of such technologies into the healthcare system.

**Abbreviations**
HF: heart failure
KCCQ: Kansas City Cardiomyopathy Questionnaire

## 1 INTRODUCTION

Heart failure (HF), a complex syndrome resulting in impaired ventricular function, is a significant cause of morbidity, mortality, and hospitalization in the United States. With over half a million new cases diagnosed each year, prevalence is growing and improvement in patient outcomes has plateaued, with 30‐day readmission rates of up to 25% and 5‐year mortality rates of 50%.^1^, ^2^

Pharmacologic guidelines for the treatment of HF include the use of angiotensin covertine enzyme inhibitors, angiotensin II receptor blockers, aldosterone antagonists, and beta‐blockers.^2^, ^3^ Recent updates also include the use of angiotensin‐neprilysin inhibitors and sodium‐glucose cotransporter‐2 inhibitors, particularly for HF patients with reduced ejection fraction.^4^ Although clinical trials have shown current treatments may reduce all‐cause and/or cardiovascular‐related mortality, renal outcomes, and HF‐related hospitalizations, the effectiveness of these interventions in the population at large is hampered by slow uptake and inadequate medication adherence.^5^, ^6^, ^7^, ^8^, ^9^

Patient self‐management, recommended by American College of Cardiology/American Heart Association (AHA) guidelines, is an effective management tool in chronic conditions.^3^, ^10^, ^11^ This may involve methods to improve medication adherence, practiced behavioral changes, and active engagement in symptom recognition and education. Interventions improving medication adherence have reduced readmission and mortality rates among the HF population.^12^ However, HF patients have historically poor self‐management, even with specialist support and social networks.^13^, ^14^ Noncompliance with medication and lifestyle modifications is common among older HF patients and contributes to disease exacerbation.^15^, ^16^, ^17^ Lack of health literacy is a major source of noncompliance, shown to be an independent risk factor for both mortality and hospitalization.^18^, ^19^, ^20^ Additional comorbidities leading to high degrees of polypharmacy also contribute to nonadherence.^21^, ^22^, ^23^ Combined, this data highlights the need for novel strategies that improve HF patient engagement and self‐care.

The use of digital health technologies has the potential to enhance and personalize care and improve the patient–clinician relationship.^24^ There are many potential benefits to both the patient and provider. Clinicians can gain access to inter‐visit clinical data that may guide treatment and allow for earlier identification of clinical decompensation. Patients may experience improved health literacy, self‐monitoring, and compliance, and become empowered to take a more active role in their care. Overall, digital health technology has the potential to enhance personalized care, increase clinical efficiency, and reduce resource utilization.

Although digital health interventions are promising, challenges of clinical workflow, physician burden, and usability must be addressed.^25^ User‐friendly platforms that both encourage patient engagement and provider adoption are essential to the success of digital interventions, highlighting the need for comprehensive randomized clinical trials that address the effects of digital health on clinical outcomes, patient usability, and clinical integration.^26^, ^27^

Our pragmatic randomized controlled trial evaluates the efficacy of three distinct digital health technologies versus usual care in improving quality of life (QOL) for patients with HF: a “smart” scale (Bodyport), an automated conversational platform (Conversa), and a coaching application (Noom). The three technologies differ fundamentally in design, user interface, and data collected. However, the use of a common control group allows for the evaluation of multiple potential interventions in an efficient trial design space. Thus, this trial will provide insight into which types of technologies can be best integrated into clinical practice and patient experience.

The primary outcome will measure the change in QOL as assessed by the Kansas City Cardiomyopathy Questionnaire (KCCQ), whereas secondary outcomes will assess clinical outcomes and efficiency, as well as patient usability and perception.

We hypothesize that digital health interventions will improve QOL of HF patients through one or both of two potential mechanisms. Digital health interventions that track symptoms and detect early warning signs of disease exacerbation, which are then relayed to providers, may result in medication and/or lifestyle adjustments that can mitigate symptoms and thus improve patient QOL. Second, technologies that allow for self‐tracking of symptoms and that provide education regarding a condition and appropriate self‐care may result in enhanced patient empowerment, medication adherence, and increased involvement of the patient in their own care management. Consistent encouragement and self‐care prompts may lead patients to make better daily lifestyle choices and more frequently communicate concerns with providers, both of which may lead to fewer symptoms and thus improved QOL.

## 2 METHODS AND ANALYSIS

This pragmatic study is an unblinded, four‐arm, parallel‐group, randomized controlled trial, to determine the efficacy of three digital health technologies versus usual care in improving QOL for patients with HF. This study will assess clinical outcomes, clinical efficiency and burden, and patient usability and perception. The trial is conducted under approval of the Yale University Institutional Review Board and adheres to the principles of the Declaration of Helsinki. The trial is registered at clinicaltrials.gov (NCT04394754).

### 2.1 Participants

This study will enroll patients actively managed by outpatient HF clinics located throughout the Yale‐New Haven Health system that follow patients closely for optimization of care and capture a socioeconomically diverse patient population.

Patients must have a current diagnosis of HF without regard to ejection fraction. Eligibility is restricted to those 18–79 years of age, as the pediatric population requires unique management and elderly patients may not benefit from digital intervention due to lack of digital literacy or advanced disease.^28^

### 2.2 Exclusion criteria

This study will exclude patients with an advanced stage of HF or comorbidities that might limit usability or benefit of the devices. To this end, patients with Class IV HF—as determined by the treating provider, who have received a heart transplant, have a ventricular assist device, are diagnosed with Stage 4 or end stage renal disease (or estimated glomerular filtration rate <30), are in hospice, are diagnosed with dementia, are determined by their physician to have a life expectancy of <6 months, are pregnant, or those otherwise unable to consent are excluded. Those in unstable living situations, or who are incarcerated, are excluded due to potential difficulties with follow‐up and the potential for shared use of devices, which is discouraged in this study. Patients currently enrolled in another digital health study will be excluded to avoid potentially contaminating effects. Finally, we will exclude patients who weigh over 400 pounds or are unable to stand for 30 seconds unassisted, to ensure that all patients can use the Bodyport scale should they be randomized to this group.

We are careful not to exclude patients on the basis of a lack of technological access. Patients will be given a prepaid smartphone with a limited data plan if they otherwise have no access to one and will be guided through email account creation if needed, as an email address is required to receive study‐related communications.

### 2.3 Interventions

Upon enrollment, patients will be randomized to one of four study arms, three of which involve the receipt of a digital health technology (see below). Patients will be encouraged, but not obligated, to engage with their technology daily. We were careful not to mandate protocols regarding frequency of use, such that we can determine real‐world use and applicability. We will emphasize to participants that no digital health technology in this study should replace usual care and will advise participants to follow the care plan outlined by their primary provider and consult with their physician or 911 during emergencies.

The three technologies included in this study were chosen through a vendor assessment process, which started with an evaluation of the market landscape for digital solutions that met essential needs of patients with Congestive Heart Failure (CHF). A competencies matrix with weighed criteria was developed and served as the basis for an initial request for information to different companies. This information included questions to inform company study operations, evidence and outcomes, patient safety, regulatory, quality, and technical requirements. Furthermore, a supplemental proof and demonstration of outcomes in CHF was requested, narrowing the final list of candidate digital solutions to 10. Bodyport, Conversa, and Noom were selected as the interventions in this study after an in‐person showcase, where all 10 solutions were evaluated by the study coordination team.

### 2.4 Bodyport

**Figure 1:**
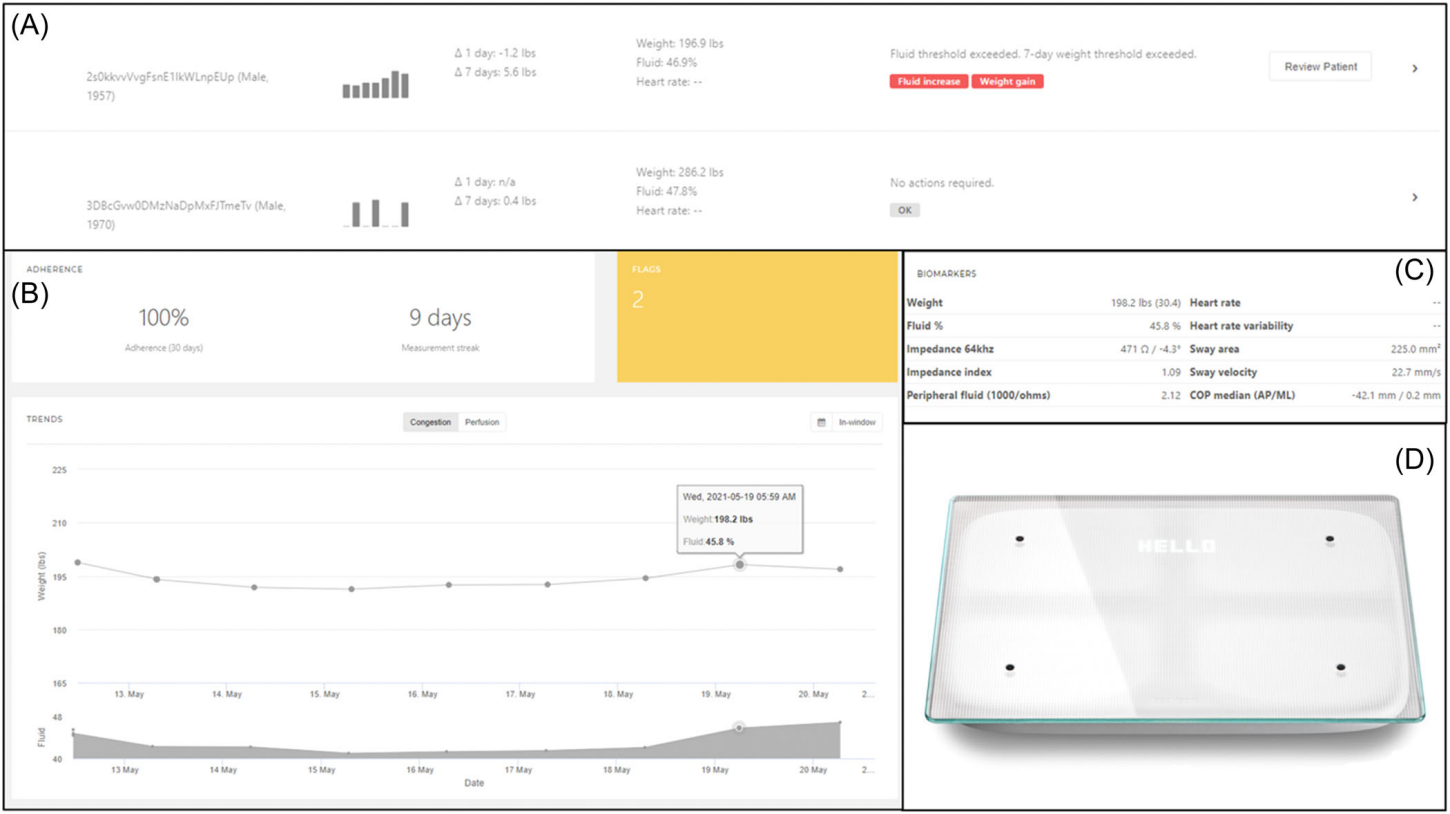
Important elements of the Bodyport physician‐facing dashboard. (A) Global dashboard displaying overall trends and alerts. (B) Detailed individual patient data showing trends in weight and perfusion. (C) Advanced metrics of cardiovascular function. (D) Representative Bodyport Cardiac Scale.

Bodyport is a data‐driven “smart” scale that measures clinically relevant markers of fluid and cardiovascular status, enabling an integrated, longitudinal assessment of congestion, and perfusion to help guide personalized HF management.^29^ Measurement data are uploaded over a cellular connection to a clinician dashboard (outside the electronic health record [EHR]) for review by both the study and clinical teams (Figure 1). Participants can also access a similar patient dashboard, to track measurements and view learning modules related to HF and effective self‐management. These modules consist of videos with transcripts, and learning checks composed of a set of multiple‐choice questions at the end. The content was created by Bodyport and is based on AHA/European Society of Cardiology (ESC) recommendations for HF education. For this study, only a subset of Bodyport biomarkers was used by the study team: body weight, impedance—a marker of fluid overload, and heart rate.

### 2.5 Conversa

**Figure 2:**
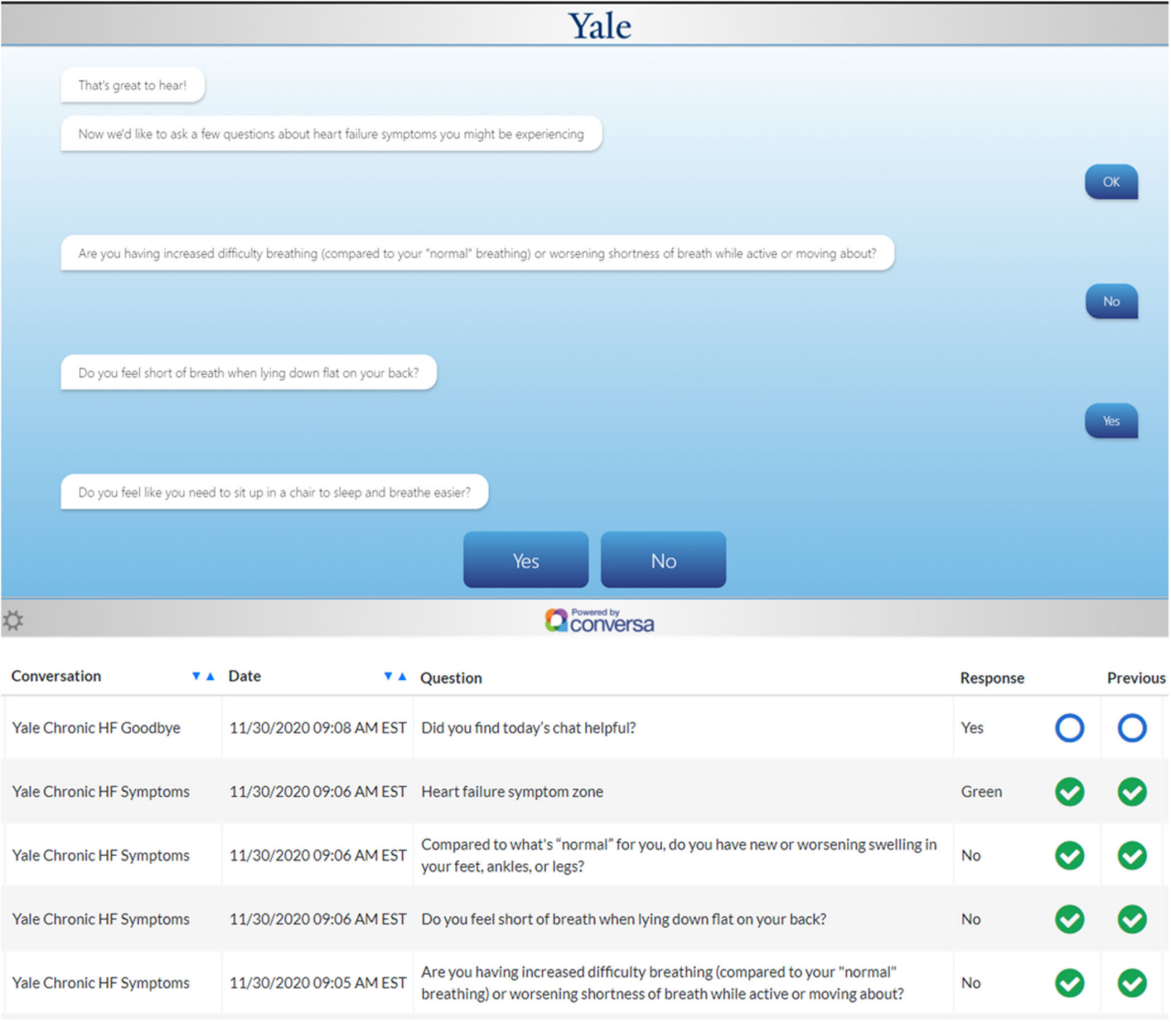
Top panel: Example “chat” for a heart failure educational module. Bottom panel: Example of the physician‐facing Conversa dashboard with a view of patient responses over time.

Conversa is an automated conversational platform designed to engage patients in self‐management. In this study, patients participate in a HF‐oriented program, where they may complete educational modules and engage in regular brief (~5 min) “clinically intelligent” chats with prescripted responses that cardiovascular physicians have vetted, to ensure appropriateness and applicability to the study population. The chats are designed to collect patient‐generated health data regarding overall health, symptoms, and self‐care, which is uploaded to a clinician dashboard that can be viewed by both the study and clinical teams (Figure 2).

### 2.6 Noom

**Figure 3:**
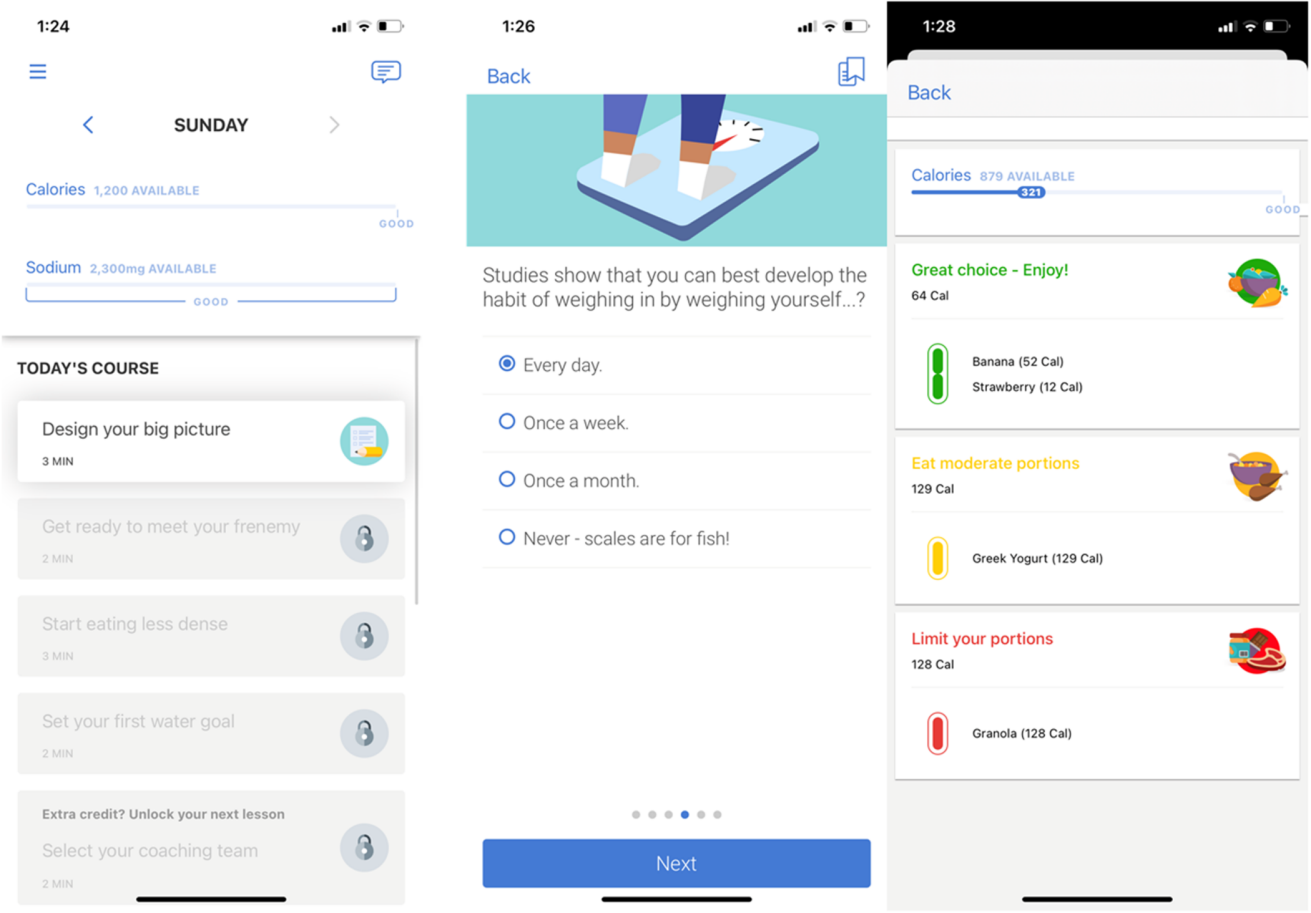
Representative Noom interface displaying daily program activities (left panel), educational programming (middle panel), and nutrient logging (right panel).

Noom is a data‐driven smartphone coaching application that uses evidence‐based behavior change techniques to coach patients towards better self‐management of their syndrome.^30^ On this HF‐tailored program, patients can track medication adherence, physical activity, symptoms, self‐reported physiological parameters (such as blood pressure and glucose levels) and eating habits via food and macronutrient logging. Live coaching support (Figure 3) and the possibility of interaction with other users through support groups within the application is also available. The Noom coaches are trained during 3 months on Noom's technology and approach, which is based on motivational interviewing and Cognitive Behavioral‐Therapy, and they are certified as lifestyle coaches by the National Board for Health and Wellness Coaching. Clinically significant changes based on patient‐reported data will be communicated to the team via a Noom coach.

### 2.7 Usual care

Patients randomized to usual care will not receive a digital health device. They will be asked to continue with their usual care as prescribed by their healthcare team. Patients will still be asked to complete all study‐related surveys and will be followed similarly as those in the intervention groups.

### 2.8 Recruitment

Eligible patients will be prospectively identified via electronic chart review. Those who meet all study criteria will either be approached during a clinic visit or contacted via phone calls, text messaging, emails, or EHR communication. Patients will be given a thorough explanation of the study and have any questions answered.

Interested patients will be guided through a web‐based enrollment platform that collects eligibility and demographic information, allows patients to read and sign an electronic consent, and directs the patient to create a study participant profile. Once this profile is complete, the patient is enrolled and is directed to their baseline surveys. All study enrollment and survey completion is performed in the VARA™ platform (Medullan).^31^

### 2.9 Randomization

Upon completion of enrollment and all baseline surveys, patients will immediately receive their assignment and any further instruction for receiving and activating any digital health technologies. Randomization is achieved through a permutated block randomization scheme, stratified by clinic, created by an independent statistician. This will ensure that case‐mix differences across study sites will not drive observed differences in technology effectiveness. Cluster randomization was not considered given the small number of clusterable entities, the heterogeneity among the clinics, which may introduce confounders, and the low risk of contamination across study arms. Randomization at the provider level was infeasible given the small number of clinicians providing care at each clinic. Additionally, patients may be cared for by multiple providers across clinic visits.

### 2.10 Blinding

This is an open label study, as the study team will be actively monitoring incoming data from participant devices to identify and properly triage red flag warnings (described below). Clinicians may also access dashboards and receive red flag warnings regarding their patients.

### 2.11 Patient experience and follow‐up

**Figure 4:**
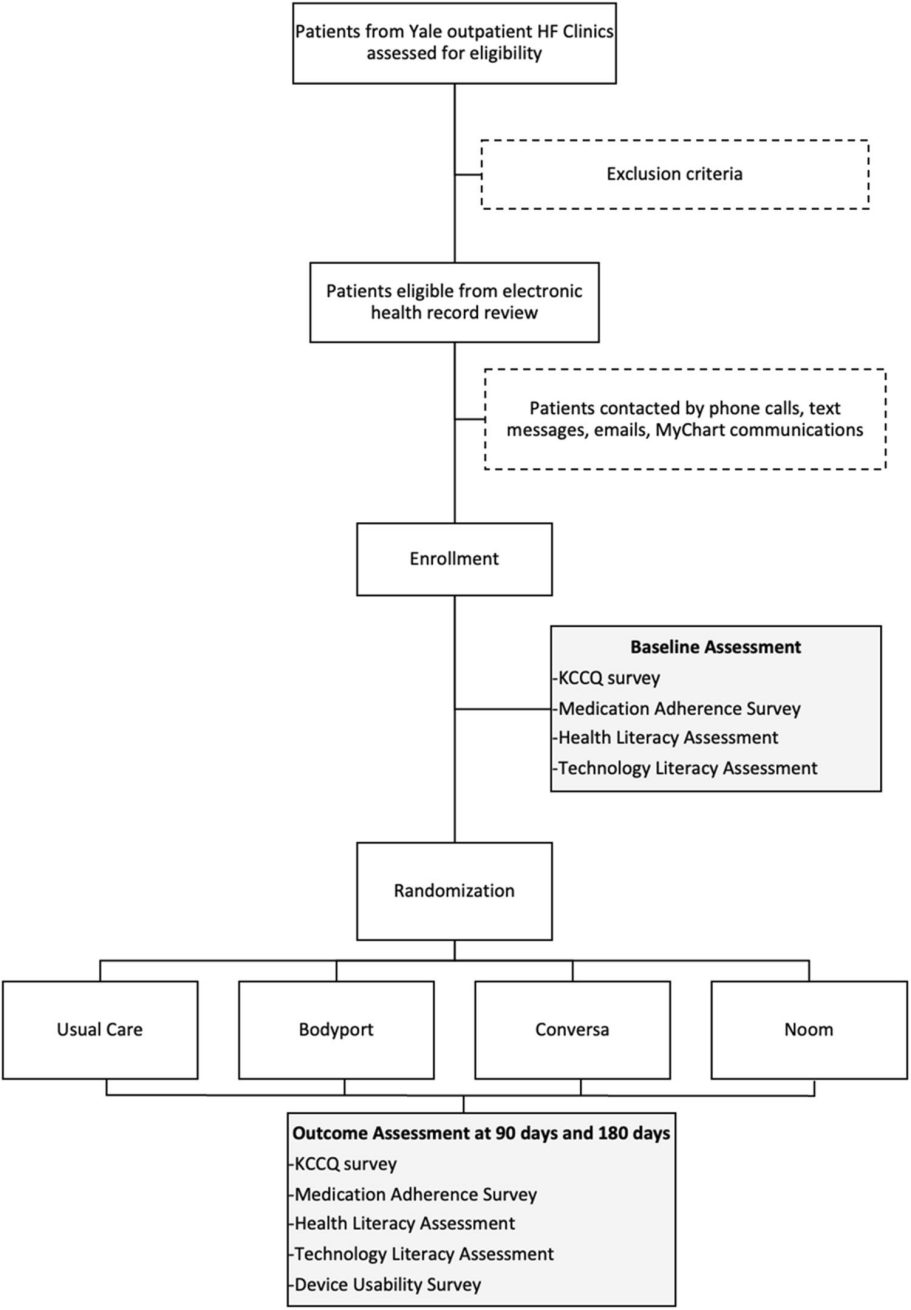
Trial profile. Patients are recruited from Yale outpatient heart failure (HF) clinics and randomized to one of three digital health interventions or usual care. They are then followed for 180 days with both clinic and telephone visits. The primary outcome is change in the Kansas City Cardiology Questionnaire (KCCQ) at 90 days.

The flow chat of the study is outlined in Figure 4. All participants will be followed for 6 months. During three remote study‐related visits at enrollment, at 3 months and 6 months, patients complete a set of surveys, including the KCCQ, a medication adherence survey (NIH PROMIS® Medication Adherence Scale),^32^ a health literacy assessment (Brief Health Literacy Screen),^33^ and a technology literacy assessment (developed in‐house). The information will be used to ascertain baseline data and track progress throughout the study in each domain.

Throughout the study, participants will be encouraged, although not mandated, to use assigned technology regularly and will continue to attend clinic visits per usual at their provider's discretion. Patient‐ and device‐generated health and usage data will be passively collected for outcome determination. During follow‐up visits, clinical data for secondary outcome determination will be collected in addition to survey completion and those randomized to an intervention arm will also be directed to complete a survey assessing usability and satisfaction with the technology.

### 2.12 Clinician experience

Conversa and Bodyport data are uploaded to EHR‐independent dashboards visible to both study and clinical teams. No standardized protocol exists regarding clinician access or use of this data so as to minimize clinician burden, with the exception of device alerts, explained below. We will emphasize to clinicians that study‐related data are supplementary to regular care and should only be used in the context of all available medical information.

### 2.13 Red flag warnings

We have prespecified several device‐specific alerts that will appear as red flag warnings in each of the technology platforms when a particular data point reaches a predetermined threshold (Table 1). For Bodyport, the research team accesses an online dashboard twice a day to identify red flag warnings; for Conversa, we receive emails when there is an alert that needs to be revised in their online dashboard; and for Noom, each of the coaches email us directly to discuss the specific red flag warnings of their participants. Each alert is vetted for clinical applicability by cardiovascular physicians on the study team and will be communicated to the clinical team via messages through the EHR platform. The patient care team will be asked, but not mandated, to follow up with the patient via a brief “check‐in” call and report any new HF symptoms and whether the patient's care plan has changed as a result. Responses are encouraged to demonstrate how these technologies alter or improve patient care. Although we recognize that specific recommendations or algorithms in conjunction with a red flag warning may be useful in eliciting changes in care by providers, our goal was to minimize clinician burden in the context of a high alert environment where alert fatigue is possible. Further, in this pilot study, we aimed to keep minimal risk to patients while assessing general efficacy and acceptance of different technologies that can be explored more deeply in future work. Thus, in the current study we provide no additional standardized protocols beyond these “check‐in” calls and leave further care to the clinical team's discretion. Additionally, should frequent alerts burden clinicians, our protocol gives flexibility to modify alerts for greatest clinical utility.

**Table 1:** Technology‐specific alerts that generate red flag warning

Technology	Alerts
Bodyport	Increase in 3 lbs over 24 h^a^ Increase in 5 lbs over 7 days^a^ Decrease in impedance of 30% over 5 days HR < 60 bpm HR > 100 bpm
Conversa	Answer “Yes” to having shortness of breath that won't go away Answer “Yes” to having to sit up to sleep and breathe easier Answer “Yes” to increased difficulty completing daily activities Answer “Yes” to having concerning symptoms (sudden chest pain, discomfort while resting) Inputs an SBP > 180 mm Hg Inputs an SBP < 90 mm Hg Inputs a DBP > 120 mm Hg Inputs a DBP < 50 mm Hg
Noom	Inputs an SBP > 170 mm Hg Inputs an SBP < 90 mm Hg Increase in 3 lbs over 24 h Increase in 5 lbs over 7 days

Abbreviations: bpm, beats per minute; DBP, diastolic blood pressure; HR, heart rate; SPB, systolic blood pressure.

^a^ Calculates the difference between the in‐window measurement today and the lowest in‐ window measurement over the past 24 h or 7 days. In‐window is 4–10 a.m. to minimize contamination by daily fluctuations in weight. Impedance, used as a marker for lower extremity edema, calculates changes in the in‐window minimum and maximum values over a 5‐day period.

### 2.14 Primary outcome

Our primary outcome is change in QOL at 3 months as measured by the KCCQ, which has shown to be responsive and sensitive at this time point.^34^ This assessment is a standardized 23‐item questionnaire, validated in multiple HF populations, which quantifies the physical limitations, frequency of symptoms, self‐efficacy, social interference, and QOL associated with living with HF. Scores range from 0 to 100, with higher scores indicating a more favorable status.

### 2.15 Secondary outcomes

Secondary outcomes of interest are listed in Table S1. Three categories of outcomes were selected as measures of successful digital health products as follows: (1) clinical outcomes, (2) clinical efficiency, and (3) usage metrics indicating patient usability, ease of use, understanding of the assigned device, and perception of engagement with health condition after the usage of technology. Each metric will be assessed at the 3‐month and 6‐month time points, to determine the stability of the effect of digital health technology over time, as clinic visits may decrease or as HF care is optimized. KCCQ scores will be reassessed at 6 months.

### 2.16 Statistical analysis

Baseline comparisons across the study arms will be conducted with χ2 tests for categorical variables, applying the Mantel–Haenszel correction for stratification by clinic site. Continuous variables will be compared using the Van–Elteren test for continuous, nonparametric variables and stratified *t* tests for normally distributed variables.

The primary analysis will be a mixed effects model comparing change in KCCQ over time in the intervention group versus the control, without accounting for a center‐by‐treatment interaction. All analyses will use the intention to treat principle. The three interventions will each be compared with control, but will not be compared with each other. In secondary analyses, the interaction with treatment centers will be modeled to determine whether certain interventions are uniquely effective in certain clinic sites.

Secondary and exploratory analyses will examine the outcomes listed above using the same procedure applied to baseline variables for outcomes assessed at one time point and using mixed‐effects models for continuous outcomes assessed at multiple time points. We will also perform an analysis of the effect of digital health interventions in general versus usual care by combining the three digital health interventions into a single analytic group.

### 2.17 Power calculations

The study was designed to provide adequate power to detect a clinically significant change in the primary outcome measure, the KCCQ score. The KCCQ composite score ranges from 0 to 100 (with 100 being the best possible score) and a change of 10 points has been determined to be the minimum clinically important difference. Within a given patient, the score is relatively stable in the absence of clinical changes, with a within‐patient standard deviation of 11.8 for patients with no change in clinical status.

To detect the minimum clinically important difference of a 10‐point change in the KCCQ, each arm of the trial must include 40 patients across all centers for 90% power at an *α*‐threshold of 0.017, giving a total of enrollment of 160. Given uncertainties around the intraclass correlation coefficient (assumed to be 0.10 here and reflecting each clinic's overall efficacy), the target sample size is inflated to 200, or 50 per trial arm. The lower *α*‐threshold represents a Bonferroni correction that provides statistical power to test multiple comparisons—each of the three digital health interventions against the control intervention. We will attempt to balance recruitment across the centers with a per‐center target of five individuals per trial arm.

### 2.18 Interim analysis

We had one interim analysis at the mid‐point of the trial (50% enrollment). This allowed us to alter the sample size or stop the trial earlier for ethical considerations, unexpected adverse events, or high efficacy if a *p* threshold of <0.0006 was reached. However, this threshold was not reached and the trial continue with further enrollments. No changes were made to the trial after the interim analysis and the threshold *p* for statistical significance at the end of the trial was stablished to be 0.016.

### 2.19 Preliminary findings: Recruitment challenges

At the time of manuscript submission, we have successfully recruited 182 patients and are conducting follow‐up visits and data collection, illustrating the feasibility of our proposed methodology. Although feasible, recruitment of the population of interest was not without its challenges, which were overcome through a series of learnings and adaptations that may prove useful for future studies.

This study was designed to be fully remote, with an online enrollment platform and digital technologies that patients can use from home. A remote design has obvious advantages in today's current climate amongst a global pandemic, when remote doctor's visits and social distancing have become the norm and appeals to patients who already feel burdened by many in‐person clinic visits.

This platform was carefully designed for ease of use and was put through an extensive user acceptance testing period to identify flaws. Despite its benefits, a fully remote design has revealed the large technological gap our target population experiences, presenting a challenge for those less technologically savvy. Eligible patients are often elderly and reside in areas of lower socioeconomic status, both demographics that are affected by the digital divide.^35^, ^36^ The development of a mobile‐ and user‐friendly platform proves essential in reducing these enrollment barriers in this and future studies. Further, some patients are reluctant to provide personal information or click links sent by unknown coordinators. Many are simply difficult to reach by phone. Our study design has undergone several adaptations to enhance user experience and improve remote recruitment. Multiple adjustments were made to the enrollment website to reduce user error and improve website navigation, and coordinators were available to fully guide participants through the online enrollment process, completing questions on behalf of the participant via the phone if necessary. The use of prescripted text messages and MyChart communications were used to make patients primed for receiving study‐related recruitment calls. Further, use of a HIPAA‐compliant dialer was used for customization of caller ID to a local area code.

Although we recognize that, because of our exclusion of those patients over 80 years of age, this study may be intrinsically biased towards those with more digital literacy, we hope that the digital divide we have experienced with this slightly younger population of HF patients and the subsequent adjustments and learnings will guide future work with digital health technologies in similar, and perhaps older, populations.

## 3 DISCUSSION

Digital health technology has proven an effective modifier of patient behavior in conditions such as mental illness, diabetes, and atrial fibrillation.^37^, ^38^, ^39^, ^40^ In the last 5 years, clinical trials have assessed diverse remote monitoring systems and their impact in the HF population, finding discrepant results.^41^, ^42^, ^43^ In this case, our goal is to evaluate the efficacy of three noninvasive and distinct technologies that may directly affect patient QOL, behavior, and engagement with their providers.

For any digital technology meant to affect patient behavior, user experience is a key design—despite collecting useful data, digital technology provides little value unless used regularly by patients or clinicians. Historically, patient retention rates of digital health technologies are low due to a lack of user‐centered design processes.^27^, ^44^ Within the HF domain, it is particularly essential to deliver user‐friendly strategies and functional capabilities that cater to an elderly population.^45^

The use of digital health technologies in the healthcare space has the potential to improve overall patient care and strengthen the provider‐patient relationship. Improvement of patient engagement and health literacy may lead to better self‐care habits and treatment compliance, while technology may allow clinicians to better identify disease exacerbation and better personalize care. A variety of primary and secondary study endpoints will address three major contributors to the success of any digital health technology: usability, clinical integration, and effect on patient outcomes. Further learnings will illustrate strategies to better engage a less technologically‐savvy population of HF patients, ensuring that this patient group is not excluded from potentially beneficial digital health solutions.

## CONFLICT OF INTERESTS

Francis P. Wilson: Founder of Efference, LLC, a medical communications company. Receives support from NIH R01DK113191, R01HS027626, P30DK079310. Nihar Desai: Under contract with the Centers for Medicare and Medicaid Services to develop and maintain performance measures used for public reporting and pay for performance programs. Reports research grants and consulting for Amgen, Astra Zeneca, Boehringer Ingelheim, Cytokinetics, MyoKardia, Relypsa, Novartis, and SCPharmaceuticals.

## Supporting information

Supporting information.

## Data Availability

Data sharing not applicable to this manuscript as no data sets were generated or analyzed during the current study.
