# Supplementary material for: Pragmatic randomized trial assessing the impact of digital health technology on quality of life in patients with heart failure: Design, rationale and implementation
Source: Clin Cardiol. 2022 Jul 12;45(8):839–49. doi: 10.1002/clc.23848 (PMC9346973; doi:10.1002/clc.23848)
Supplement: Supplementary file 1 — Supporting information. [file CLC-45-839-s001.docx]

| **Secondary Outcomes** | |
| --- | --- |
| **Clinical Outcomes** | ED visits  Hospital admissions  Rates of guideline directed medical therapy  AKD development  Clinic no-show rates  Mortality |
| **Clinical Efficiency** | Number of in-person clinic visits  Number of virtual/phone clinic visits  Number of remote device checks  Number of calls made to clinic with question/inquiry for provider  Number of calls made by provider/clinic to patient for check-in  Number of calls made by provider/clinic to patient with test results  Number of appointment reminder calls made to patient |
| **Usage metrics** | Number of enrolled patients  Mean interactions with technology per week  Proportion of interactions by median participant  Satisfaction with device/technology  Content rating  Perceived impact |

ED: Emergency department, AKD: Acute kidney disease

**Supplemental Table.** Secondary Outcomes
